# Clostridioides difficile recovered from hospital patients, livestock and dogs in Nigeria share near-identical genome sequences

**DOI:** 10.1099/mgen.0.001342

**Published:** 2025-01-30

**Authors:** Emmanuel O. Ngbede, Vera Junker, Baban Kolte, Martinique Frentrup, Judith Boldt, Warren N. Fawley, Mark H. Wilcox, Ed J. Kuijper, Wiep Klaas Smits, Ulrich Nübel

**Affiliations:** 1 Leibniz Institute DSMZ - German Collection of Microorganisms and Cell Cultures, Microbial Genome Research, Braunschweig, Germany; 2 Department of Veterinary Microbiology, Federal University of Agriculture, Makurdi, Nigeria; 3 Technical University Braunschweig, Institute of Microbiology, Braunschweig, Germany; 4 German Center for Infection Research (DZIF), Partner Site Braunschweig-Hannover, Braunschweig, Germany; 5 School of Medicine, Leeds Teaching Hospitals and University of Leeds, Leeds, UK; 6 Leiden University Medical Center, National Expertise Center for C. difficile Infections, Leiden, Netherlands

**Keywords:** Africa, *Clostridioides difficile*, epidemiology, One Health, phylogenomics, zoonosis

## Abstract

Genomic data on *Clostridioides difficile* from the African continent are currently lacking, resulting in the region being under-represented in global analyses of *C. difficile* infection (CDI) epidemiology. For the first time in Nigeria, we utilized whole-genome sequencing and phylogenetic tools to compare *C. difficile* isolates from diarrhoeic human patients (*n*=142), livestock (*n*=38), poultry manure (*n*=5) and dogs (*n*=9) in the same geographic area (Makurdi, north-central Nigeria) and relate them to the global *C. difficile* population. In addition, selected isolates were tested for antimicrobial susceptibility (*n*=33) and characterized by PCR ribotyping (*n*=53). Hierarchical clustering of core-genome multilocus sequence typing (cgMLST) allelic profiles revealed large diversity at the level HC150 (i.e. clusters of related genomes with maximally 150 pairwise allelic differences), which was previously shown to correlate with PCR ribotypes (RT). While several globally disseminated strains were detected, including HC150_1 (associated with RT078), HC150_3 (RT001) and HC150_3622 (RT014), 42 HC150 clusters (79%) represented unique genotypes that were new to the public genomic record, and 16 (30%) of these were novel PCR ribotypes. Considerable proportions of the *C. difficile* isolates displayed resistance to fluoroquinolones, macrolides and linezolid, potentially reflecting human and animal antibiotic consumption patterns in the region. Notably, our comparative phylogenomic analyses revealed human–human, human–livestock and farm–farm sharing of near-identical *C. difficile* genomes (≤2 core-genome allelic differences), suggesting the continued spread of multiple strains across human and animal (pig, poultry, cattle and dog) host populations. Our findings highlight the interconnectivity between livestock production and the epidemiology of human CDI and inform the need for increased CDI awareness among clinicians in this region. A large proportion of *C. difficile* strains appeared to be unique to the region, reflecting both the significant geographic patterning present in the *C. difficile* population and a general need for additional pathogen sequencing data from Africa.

## Data Summary

Raw sequence read files for all isolates have been deposited in the National Center for Biotechnology Information (NCBI) under the project accession number PRJNA1092862. In addition, genome assemblies of the isolates reported in this study are available in EnteroBase (https://enterobase.warwick.ac.uk/species/index/clostridium). NCBI accession numbers and EnteroBase assembly barcodes for each isolate are listed in Table S1, sheet 1.

**Impact Statement**

Using bacterial genome sequencing, we investigated the spread of the pathogen *Clostridioides difficile* among diarrhoeic hospital patients in Makurdi (Nigeria), and livestock (pigs, poultry and cattle) and dogs in the same area. The sharing of multiple near-identical genome sequences suggested frequent transmission of the pathogen between the different sectors, indicating its zoonotic potential. A large proportion of *C. difficile* strains were new to the public genomic record, highlighting a general lack of pathogen genomic data from Africa.

## Introduction

*Clostridioides difficile* infection (CDI) is often associated with high morbidity and mortality [1]. The disease has varying clinical presentations including mild diarrhoea, pseudomembranous colitis or toxic megacolon and death. It is caused by the effects of *C. difficile* toxins on the colonic epithelium [2], including toxins A and B (encoded by the genes *tcdA* and *tcdB*), and in some strains, the additional binary toxin (*cdtA/B* genes).

Due to its clinical significance, *C. difficile* has increasingly been the focus of surveillance and molecular epidemiological studies. However, unlike countries in Europe, Asia, North America and Australia, where considerable progress has been made in understanding and describing its epidemiology and transmission dynamics*,* the situation on the African continent, which bears one of the highest burdens of diarrhoea, is poorly understood [3,5]. In Africa, CDI is rarely considered a differential cause of diarrhoea, largely due to a low index of clinical suspicion and limited diagnostic capabilities [3,6]. The few studies carried out on the African continent have focused on PCR ribotyping and toxin detection and have detected the pathogen in both humans and animals. The majority of these studies have reported a predominance of non-toxigenic strains, in contrast to other parts of the globe [3,6,18]. Several studies have highlighted the presence of *C. difficile* in humans [16,17] and dogs [18] in Nigeria.

Reports from Europe, North America and parts of Asia have shown the power of whole-genome sequencing (WGS) in providing insights into the epidemiology and transmission dynamics of CDI [19,24]. The potential for zoonotic transmission of *C. difficile* has been demonstrated by genetic overlap between human and animal strains [25]. However, *C. difficile* genome sequences from Africa are underrepresented in public databases [22,25,27]. Of the more than 30 500 publicly available *C. difficile* genome datasets hosted in EnteroBase (as of June 2024), only 47 (0.15%) are from Africa, specifically from South Africa [28]. In addition to creating a surveillance blind spot for understanding global *C. difficile* population genomics, this underrepresentation may promote the erroneous assumption that CDI may not be a problem on the African continent, further lowering clinical suspicion and prioritization of the disease.

*C. difficile* was recovered from the faeces of livestock and dogs repeatedly, and these animals have been suspected as potential reservoirs and sources of the pathogen based on closely related *C. difficile* genome sequences [22,26, 29]. In Nigeria, backyard farming is widespread and characterized by the rearing of poultry and pigs within or in close proximity to human dwellings [30,31]. For example, it is common practice for some farmers to allow their pigs to roam freely in human dwellings. Direct and frequent contact between humans and animals increases the likelihood of bidirectional exchange of pathogens. Farmers are also known to dispose of untreated litter from their farms into public water bodies or use it for fertilization of agricultural land, thereby contaminating the environment and exposing the unsuspecting public to associated pathogens [32,34]. Environmental contamination with viable *C. difficile* resulting from the application of manure for fertilization has been reported [22]. In Nigeria, poor to non-existent biosecurity measures on farms, particularly the practice of farm workers visiting other farms without proper decontamination and the procurement of animals from open markets, may facilitate the spread of the pathogen from one farm to another [35]. Currently, there is no genomic information available that would help to understand the transmission dynamics in this setting and contextualize the potential risks.

Our objective in the present study was to investigate the occurrence and genomic epidemiology of *C. difficile* among humans and animals in Makurdi, north-central Nigeria. To this end, we have recovered *C. difficile* strains from diarrhoeic human hospital patients and from cattle, pigs, poultry, poultry manure and dogs. All samples were collected in the city of Makurdi, north-central Nigeria. WGS and phylogenomic analyses allowed us to describe the *C. difficile* population structure, infer plausible clonal spread among humans and animals and relate our data to the large body of publicly available genome sequences.

## Methods

### Study design and sampling

**Fig. 1.**
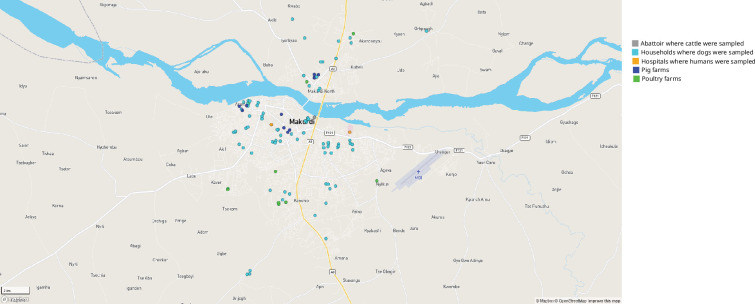
Open Street Map of the study location. Sampling points are coloured based on the sample source, i.e. abattoir (cattle), households (dogs), hospitals (human), pig farms (pigs) and poultry farms (poultry and manure).

Using a cross-sectional approach, faecal samples were collected between January 2021 and December 2022 to recover *C. difficile* from diarrhoeic (defined as ≥3 loose stools within 24 h [36]) humans in two tertiary hospitals (note that CDI is not often considered nor diagnosed in these settings). Samples were collected from all diarrhoeic patients who were willing to participate in the study. Additional faecal samples were collected from dogs in households and animals in backyard farms and in a slaughter facility. Manure samples were additionally collected from poultry farms. The study setting was Makurdi Local Government Area, in Benue State, north-central Nigeria, with a relatively dense human and animal population (housed or freely roaming in close proximity to human dwellings), providing increased opportunities for potential bidirectional spillover of pathogens between humans and animals. All sampling sites and sources including farms, slaughterhouses, households (for dogs) and hospitals were located within the area and a distance of <15 km from each other (Fig. 1). The subjects were enrolled based on a convenience sample technique, i.e. located within the geographic location and willingness to participate by means of oral consent from diarrhoeic individual (or guardian), dog owner(s) and management of the farm/slaughter facility. No restriction was placed on the type or scale of farm operation; however, the majority was smallholder backyard farms. Up to 10 animals were sampled in each farm, while all dogs in a household were sampled. A total of 2201 samples originating from 1630 humans, 120 poultry and 20 manures from 12 poultry farms, 130 pigs from 13 pig farms, 84 cattle from 1 abattoir and 217 dogs from 92 different households were collected.

### Cultivation and recovery of *C. difficile*

Isolation of *C. difficile* from the respective samples was carried out using a previously described protocol [22]. Briefly, approximately 1 g of faecal matter or manure was inoculated directly into 10 ml of pre-reduced brain heart infusion broth (Roth, Germany) supplemented with 0.1% taurocholic acid (Sigma, Germany), 0.1% cysteine (Sigma, Germany) and *C. difficile* selective supplement (Oxoid, UK) in Hungate tubes. The tubes were incubated at 37 °C for a period of 7 days. After the period of incubation, ethanol shock was performed by adding 500 µl of absolute ethanol to an equal volume of the culture, and the mixture was incubated on a shaker for 1 h at room temperature to kill vegetative cells (but not *C. difficile* spores). Following this, the mixture was centrifuged at 2500 ***g*** for 5 min, and the resulting cell pellet was resuspended in 100 µl PBS. One hundred microlitres of the resuspended pellet were then plated on Oxoid *C. difficile* agar supplemented with d-cycloserine and cefoxitin (Oxoid, UK) and incubated at 37 °C under anaerobic conditions (using an anaerobic jar and an Oxoid anaerobic gas generator) for 72 h. Colonies on the plates with typical *C. difficile* morphology (grey, flat colonies with irregular margins) were further streaked onto ChromID agar (bioMérieux) for identification and sub-cultured to purity on blood agar plates, followed by PCR confirmation of the isolates using previously described primers [37].

### WGS and bioinformatics analysis

Genomic DNA was extracted from subcultures of *C. difficile* isolates using the DNeasy Blood and Tissue kit (Qiagen, Germany) and quantified using the Qubit 3.0 fluorometer (Life Technologies). Sequencing libraries were prepared using the miniaturized Nextera XT-protocol [38] and sequenced on the NextSeq2000 sequencer (Illumina, USA) to generate 150 bp paired-end reads. The sequence reads were then uploaded to the *Clostridioides* database of the EnteroBase platform (http://enterobase.warwick.ac.uk/), which uses a consistent pipeline for assembling reads into draft genomes and predefined quality control metrics to exclude assemblies of poor quality and those not classified as *C. difficile* [21]. Genome assemblies were genotyped using core-genome multilocus sequence typing (cgMLST), and cgMLST-based hierarchical clustering within EnteroBase was performed. Hierarchical clustering at level HC150 (maximally 150 cgMLST allelic differences between pairs of genomes) was used to predict PCR ribotypes [21,39]. Genetic relatedness at levels HC2 and HC0 identified closely related or indistinguishable core genomes, respectively [21]. Genetic relatedness was visualized using phylogenetic rapid neighbour-joining (NJ) trees calculated with GrapeTree as implemented in EnteroBase [21,40]. The SNP analysis tools implemented within EnteroBase were used to call non-repetitive SNPs [41] against the reference DSM1296^T^ genome (NCBI RefSeq assembly GCF_001077535.1, clade 1, ST3, RT001) and to generate a SNP-based phylogenetic tree for the cryptic clades, which was then visualized using iTol [42]. The rapid NJ and SNP trees were further annotated in Inkscape version 1.3.2 (www.inkscape.org/).

### Analyses of SNPs

Genome-wide SNPs were identified by using the nf-core/BactMap pipeline (https://nf-co.re/bactmap/1.0.0) version 1.0.0 using the default settings and the following parameters: -- genome_size, --remove_recombination and --trim turned on [43]. All reads were mapped to the reference genome NZ_CP016318.1 (clade 1, ST54, RT012). The nf-core/BactMap utilizes BWA mem for read mapping, SAMtools for sorting and index alignment, Bcftools for variant call and filtering, SNP-sites for extracting variant sites and Gubbins for removing recombination. The program snp-dists version 0.8.2 (https://github.com/tseemann/snp-dists) was employed to generate the SNP pairwise distance matrix from the recombination-free alignment.

### Determination of average nucleotide identity (ANI)

We calculated the genome-wide ANI for our genome sequences in comparison to the *C. difficile* type strain DSM1296^T^ (NCBI RefSeq assembly GCF_001077535.1) using the program fastANI version 1.33 with default parameters [44].

### Toxin gene detection

The presence of toxin genes (*tcdA*, *tcdB*, *cdtA* and *cdtB*) and their corresponding regulatory genes (*cdtR*, *tcdR*, *tcdC* and *tcdE*) was investigated within EnteroBase by *in silico* scanning of the genomes for alleles of the different toxin gene loci as previously described [22].

### Antimicrobial susceptibility and detection of genetic resistance determinants

Assembled genomes were downloaded from EnteroBase and queried for the presence of antimicrobial resistance determinants using AMRFinder Plus version 3.12.8 [45]. We additionally scanned the genomes for the presence of genetic determinants of resistance to fidaxomicin, vancomycin and metronidazole by using local blastn and tblastx search (blast version 2.14.0), with the sequence identity and coverage thresholds set at 95 and 80%, respectively [46].

Susceptibility of *C. difficile* isolates to clindamycin, vancomycin, tetracycline, moxifloxacin, rifampicin, linezolid and ciprofloxacin was measured by gradient diffusion using MIC strips (Liofilchem, Italy). The MIC strips were placed on *Brucella* agar (supplemented with 5% horse blood) plates pre-inoculated with 100 µl *C*. *difficile* suspensions at 0.5 McFarland and incubated at 37 °C for 48 h under anaerobic conditions. MIC was defined as the MIC value (µg/ml) on the strip at the point of intersection of the inhibitory zone with the strip. Susceptibility to metronidazole was measured by the agar dilution method [47]. Except for linezolid, MIC breakpoints for resistance were interpreted according to European Committee on Antimicrobial Susceptibility Testing (EUCAST) guidelines (https://www.eucast.org/clinical_breakpoints) and the Clinical Laboratory and Standards Institute (CLSI) guidelines [48].

### PCR ribotyping

Assignment of PCR ribotypes to selected isolates within EnteroBase was carried out by capillary PCR ribotyping using a previously described protocol at the Dutch National Expertise Center for CDIs [49]. If no PCR ribotype could be assigned there, fragment files (*.fsa) were shared with the UK Health Security Agency *C. difficile* laboratory at Leeds Teaching Hospitals NHS Trust, which maintains the reference database for PCR ribotypes.

## Results

### *C. difficile* was present in hospitalized patients, livestock and dogs in Nigeria

Two hundred and twenty-eight *C. difficile* isolates originating from humans (*n*=164), pigs (*n*=27), poultry (*n*=16), dogs (*n*=13), poultry manure (*n*=5) and cattle (*n*=3) were cultivated and whole-genome sequenced (Table 1). Human isolates were successfully recovered from only one of the two study hospitals. In only six human stool samples (2.6%, 6/228), we detected a co-occurrence of *C. difficile* and other potential diarrhoeagenic pathogens, i.e. extended spectrum β-lactamase *Escherichia coli* (*n*=5) and *Salmonella enterica* (*n*=1). Dog isolates were recovered from samples from 13 different households, while pig and poultry isolates were from seven and six different farms, respectively. Consistent with previous reports from the African continent [16,50, 51] and in contrast to other geographic regions, *C. difficile* was predominantly recovered from relatively younger individuals. The overall median age was 26 years (*n*=164, range: 4–72, mean: 30.4) for all *C. difficile*-positive individuals and 27.5 years (*n*=35, range: 9–48, mean: 26.5) for humans with toxin-positive strains, respectively.

**Table 1.** Sources of *C. difficile* isolates

Sources	Number of			
	Samples collected	Isolates recovered	Toxin-positive isolates	Genomes analysed
Cattle	84	3	0	2
Dog	217	13	2	9
Pig	130	27	11	22
Poultry	120	16	6	14
Poultry manure	20	5	2	5
Human	1630	164	14	142
Total	2201	228	35	194

### cgMLST revealed a large diversity of *C. difficile*

**Fig. 2.**
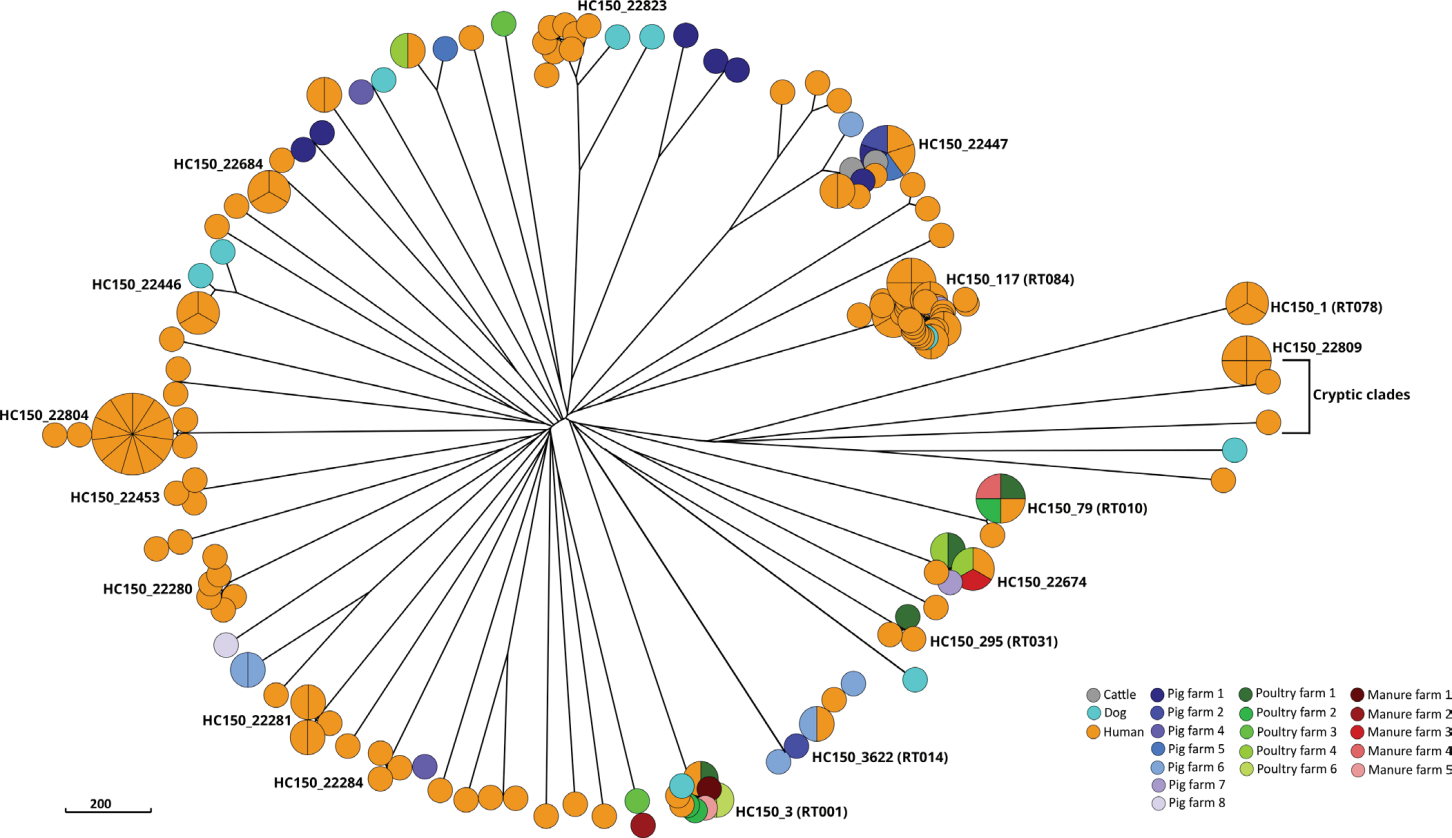
A rapid neighbour-joining phylogenetic tree based on cgMLST allelic distances. Nodes are coloured by isolation sources, and HC150 clusters are indicated for clades with ≥3 isolates. The scale bar indicates the branch length corresponding to 200 cgMLST allelic differences.

One hundred ninety-four genome sequences (85%) passed the EnteroBase quality criteria and were thus available for further analyses. Hierarchical clustering assigned these genome sequences into 53 clusters at level HC150 (previously shown to correlate with PCR ribotypes [21]), indicating a genetically diverse *C. difficile* population (Figs 2 and Supplementary Material 1 available in the online Supplementary Material). HC150 diversity was larger for human isolates (*n*=41) compared to animal isolates (*n*=23), even after normalization for the number of isolates from each source. Eleven HC150 clusters comprised genomes from several different host species (Fig. 2). Across all sources, we observed a dominance of non-toxigenic *C. difficile*, particularly HC150_117 (associated with RT084; Figs 2 and Supplementary Material 1) which is a frequently recovered strain in human and animal studies across Africa [8,10, 14, 18, 52]. Isolates in two additional HC150 clusters (HC150_22674, HC150_22679) displayed RT084, and closer inspection revealed that they were related at level HC200 (HC200_22674; Table 2). Globally disseminated HC150 complexes included HC150_1 (RT078/126), HC150_3 (RT001) and HC150_3622 (RT014) (Fig. 2). Notably and congruent to previous observations from the African continent, the epidemic HC150_4 (RT027) was not detected in this study.

**Table 2.** PCR ribotypes for 53 isolates, selected on the basis of their unique HC150 assignments

Isolate ID	HC150	Toxin genes	Host	PCR ribotype determined by reference lab
CD-22–00751	HC150_79	–	Human	RT010
CD-22–00753	HC150_22284	–	Human	RT681
CD-22–00879	HC150_22438	–	Human	RT1132 (novel)
CD-22–00881	HC150_22431	–	Human	RT1133 (novel)
CD-22–00882	HC150_22442	–	Human	RT130
CD-22–00950	HC150_22280	–	Human	RT253
CD-22–01469	HC150_22282	–	Human	RT369
CD-22–01471	HC150_22432	–	Human	RT307
CD-22–01473	HC150_22279	*tcdA, tcdB*	Pig	RT718
CD-22–01510	HC150_22434	*tcdA, tcdB*	Pig	Novel‡
CD-22–01541	HC150_19890∗	*tcdA, tcdB*	Pig	RT718
CD-22–01546	HC150_16931∗	–	Dog	RT113
CD-22–01558	HC150_22441	–	Pig	RT1134 (novel)
CD-22–01581	HC150_1352	–	Dog	RT051
CD-22–01614	HC150_22457	*tcdA, tcdB*	Pig	RT1135 (novel)
CD-23–00006	HC150_22447	–	Pig	RT1137 (novel)
CD-23–00011	HC150_3622	*tcdA, tcdB*	Pig	RT014
CD-23–00014	HC150_22452	–	Pig	RT244
CD-23–00015	HC150_22818	*–*	Pig	RT1138 (novel)
CD-23–00370	HC150_22430	–	Human	RT713
CD-23–00372†	HC150_22679	–	Human	RT084
CD-23–00373	HC150_295	–	Human	RT031
CD-23–00379	HC150_22453	–	Human	RT011
CD-23–00380	HC150_127∗	*tcdA, tcdB*	Human	RT848
CD-23–00382	HC150_22697	–	Human	Novel‡
CD-23–00390	HC150_22684	–	Human	RT011
CD-23–00399	HC150_22687	–	Human	Novel‡
CD-23–00400	HC150_1	*tcdA, tcdB, cdtAB*	Human	RT078
CD-23–00401†	HC150_117	–	Human	RT084
CD-23–00408	HC150_23556	–	Human	RT1140 (novel)
CD-23–00411	HC150_187	*tcdA, tcdB*	Human	RT054
CD-23–00412	HC150_22693	–	Human	RT573
CD-23–00413	HC150_22690	–	Human	RT253
CD-23–00415	HC150_22675	–	Human	RT1059
CD-23–00419	HC150_3	*tcdA, tcdB*	Human	RT001
CD-23–00544	HC150_22446	–	Human	RT535
CD-23–00554	HC150_22808	*tcdA, tcdB*	Human	RT943
CD-23–00561	HC150_22820	–	Human	RT1141 (novel)
CD-23–00563	HC150_22823	–	Human	RT097
CD-23–00598	HC150_22804	–	Human	RT056
CD-23–00599	HC150_22809	–	Human	RT1142 (novel)
CD-23–00600	HC150_22813	*tcdA, tcdB*	Human	RT1010
CD-23–00693	HC150_22881	–	Human	RT019
CD-23–00815	HC150_22906	–	Human	RT1143 (novel)
CD-23-00822	HC150_22456	–	Human	RT1136 (novel)
CD-23–00823	HC150_22900	–	Human	RT200
CD-23–00824	HC150_22901	–	Human	RT1144 (novel)
CD-23–00829	HC150_23580	–	Poultry	RT1145 (novel)
CD-23–00853	HC150_22674†	–	Poultry	RT084
CD-23–00861	HC150_22889	–	Human	RT535
CD-23–00862	HC150_23611	–	Human	RT761
CD-23–00866	HC150_18358∗	–	Dog	RT097
CD-23–00876	HC150_23594	–	Human	RT737

- = non-toxigenic.

∗ = had relatives in the genome database but without PCR ribotype information.

† = these isolates were related at level HC200 (HC200_22674).

‡ = novel–ribotype number assignments yet to be provided by the reference laboratory.

**Fig. 3.**
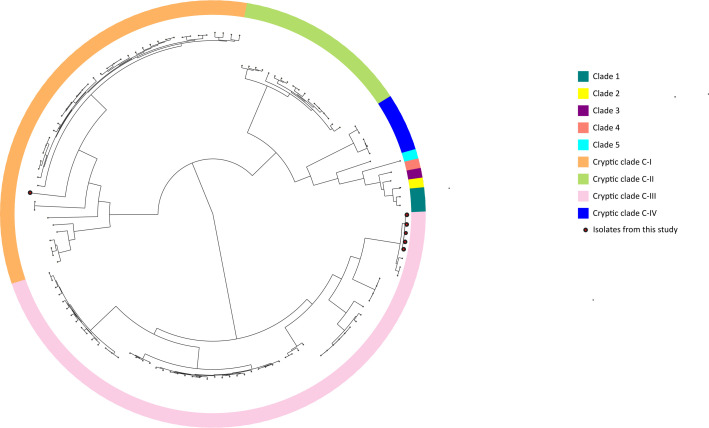
Phylogeny of *C. difficile* cryptic clades. A rapid neighbour-joining tree based on SNP variation among 137 *C*. *difficile* genomes (6 canonical and 131 cryptic clade genomes). Branch and background colours indicate the different clades as previously described [32,53, 103 ]. Red-coloured leaves represent genomes from the present study.

Six isolates originating from human samples were affiliated to hierarchical clusters HC2500_19171 (*n*=5) and HC2500_23594 (*n*=1), which correspond to 'cryptic clades' that are deeply branching from the *C. difficile* species radiation [21,29, 53] (Table S1, sheet 2). Notably, these isolates (and not any other) were colourless on the ChromID (bioMérieux) agar. Whole genome comparison to the *C. difficile* type strain revealed ANI values of 89–91 %, confirming their large phylogenetic distance to canonical *C. difficile* [53]. The 7-gene MLST identified all five HC2500_19171 isolates as triple-locus variants of *C. difficile* sequence type 889 (ST889), and PCR ribotyping results identified them as novel PCR ribotype. In the case of HC2500_23594, all seven MLST alleles appeared novel (Table S1, sheet 3), and the isolate was assigned to RT737. SNP-based phylogenetic analysis with all 119 cryptic clade genomes hosted in EnteroBase (as of June 2024) showed that HC2500_19171 isolates clustered with cryptic clade C-III and the HC2500_23594 isolate clustered with cryptic clade C-I (Fig. 3).

### Antimicrobial susceptibility, resistance and toxin gene detection

We detected both toxin genes *tcdA* and *tcdB* in 33 (17%) genomes, with three of these harbouring the binary toxin (*cdtA/B*) genes in addition. Isolates originating from pigs (41%), poultry (31%) and poultry manure (40%) had higher proportions of toxin-gene-positive genomes compared to humans (8%) and dogs (8%) (Table S1, sheet 1).

No known genetic determinants of resistance to fidaxomicin, metronidazole and vancomycin, i.e. the drugs commonly used for CDI treatment, were detected by blast analyses [46]. Based on AMRFinder queries, the *erm(B*) gene causing clindamycin resistance was the most frequently detected (*n*=54) and often co-occurred in the same genome with *tet(M*), independent from isolation sources (Table S1, sheet 1). Mutations of the gyrase genes (*gyrA* and *gyrB*) causing fluoroquinolone resistance in *C. difficile* were detected in 47 genomes originating from both humans and animals. The *gyrA*-Thr82Ile mutation was detected in a HC150_79 (RT010) genome co-harbouring the *rpoB-*H502N and *rpoB-*R505K mutations known to mediate rifamycin resistance. The *cfr(B*) and *erm(A*) genes, encoding linezolid and macrolide-lincosamide-streptogramin (MLS_B_) resistance, respectively, were detected in three genomes (Table S1, sheet 1).

Results from antimicrobial susceptibility testing of 33 isolates selected across different sources and HC150 complexes revealed concordance with the genotyping results, except that most isolates harbouring the *tet(M*) gene were susceptible to tetracycline (MIC_range_ 0.032–8 µg ml^−1^, Table 3), a phenomenon previously attributed to inducible resistance [54,55]. Regarding fluoroquinolone resistance, consistent with previous reports [56], all isolates carrying the *gyrB* mutations were resistant to ciprofloxacin but susceptible to moxifloxacin, whereas the single isolate harbouring the *gyrA*-Thr82Ile mutation was resistant to both fluoroquinolone drugs tested. We identified three HC150_1 (RT078/126) isolates recovered from humans with linezolid MICs of 16–24 µg ml^−1^ (Table 3). There is currently no CLSI or EUCAST-recommended breakpoint for the interpretation of linezolid resistance in *C. difficile*. However, a threshold that was proposed for anaerobic bacteria a while ago [57] suggests that our isolates may have acquired linezolid resistance, which would also be consistent with currently available data on linezolid MIC distributions in *C. difficile* (https://mic.eucast.org/search/; accessed 22 July 2024). Coincidently, these three isolates were the only ones in our collection that harboured the *cfr(B*) gene (Table S1, sheet 1).

**Table 3.** Antibiotic susceptibility testing results for 33 selected *C. difficile* isolates

ID	HC150	Source		MIC µg^−1^ ml^−1^ (clinical breakpoint)						
			**CLI** **∗,†** **(>8 µg ml** ^**−1**^ **)**	**VAN** **∗,‡** **(>2 µg ml** ^**−1**^ **)**	**TET** **∗,†** **(≥16 µg ml** ^**−1**^ **)**	**MOX** **∗,†** **(≥4 µg ml** ^**−1**^ **)**	**CIP** **∗,‡** **(≥8 µg ml** ^**−1**^ **)**	**LIN** **∗,§** **(>8 µg ml** ^**−1**^ **)**	**RIF** **∗,‡** **(≥16 µg ml** ^**−1**^ **)**	**MET** **∗,‡** **(2 µg ml** ^**−1**^ **)**
CD-22–00751	HC150_79	Human	>256	0.5	4	>32	>32	1.5	>32	1
CD-22–00753	HC150_22284	Human	2	0.75	0.064	1.5	16	1.5	<0.002	0.25
CD-22–00880	HC150_117	Human	>256	0.5	2	1	8	1	<0.002	0.5
CD-22–01471	HC150_22432	Dog	1	0.5	0.064	1.5	8	1.5	<0.002	0.5
CD-22–01476	HC150_22434	Pig	1	0.5	0.064	1.5	12	2	<0.002	0.5
CD-22–01493	HC150_117	Human	0.38	0.5	0.032	1.5	0.5	2	<0.002	0.5
CD-22–01496	HC150_117	Human	>256	0.75	16	2	12	2	<0.002	2
CD-22–01541	HC150_19890	Pig	0.38	0.38	0.094	1	24	8	<0.002	0.25
CD-22–01546	HC150_16931	Dog	0.75	0.75	0.064	1	>32	0.5	<0.002	0.25
CD-22–01547	HC150_22446	Dog	1.5	1	0.064	1	>32	1.5	<0.002	1
CD-22–01560	HC150_22284	Pig	2	0.75	0.094	1.5	12	1.5	<0.002	0.5
CD-22–01574	HC150_117	Dog	0.5	1	1	1	12	1.5	<0.002	1
CD-22–01609	HC150_117	Pig	–	0.75	1.5	1.5	24	1.5	–	0.5
CD-22–01622	HC150_19890	Pig	1.5	1.5	0.0064	–	6	1.5	<0.002	0.5
CD-23–00012	HC150_3622	Pig	4	0.5	8	1.5	>32	2	<0.002	0.5
CD-23–00394	HC150_117	Human	>256	0.25	6	3	>32	1.5	<0.002	1
CD-23–00400	HC150_1	Human	>256	0.5	4	0.5	4	24	–	0.25
CD-23–00403	HC150_1	Human	>256	0.38	4	–	4	16	–	0.5
CD-23–00404	HC150_1	Human	>256	0.38	8	–	6	16	<0.002	0.5
CD-23–00419	HC150_3	Human	>256	0.5	0.25	1.5	>32	3.0	<0.002	0.5
CD-23–00542	HC150_22441	Human	1.5	0.38	0.064	1	8	2	<0.002	0.5
CD-23–00550	HC150_22804	Human	8	1.5	0.047	0.75	16	2	<0.002	0.25
CD-23–00552	HC150_22441	Human	6	0.25	0.047	1	>32	1.5	<0.002	0.25
CD-23–00577	HC150_22809	Human	0.5	0.75	0.094	1	8	2	–	2
CD-23–00581	HC150_22804	Human	6	0.38	0.032	1	>32	1.5	<0.002	0.25
CD-23–00582	HC150_22446	Human	1.5	0.75	0.094	1.5	6	1.5	<0.002	0.25
CD-23–00594	HC150_3622	Human	2	0.5	8	1.5	4	2	<0.002	0.5
CD-23–00599	HC150_22809	Human	0.25	0.75	0.125	0.75	8	2	2	1
CD-23–00815	HC150_22906	Human	3	0.75	0.047	1	6	1.5	<0.002	0.25
CD-23–00837	HC150_187	Human	>256	0.75	4	1	8	2	<0.002	0.5
CD-23–00847	HC150_3	Poultry manure	12	1	0.032	0.75	>32	1	<0.002	0.25
CD-23–00869	HC150_3	Dog	8	0.19	0.064	1	16	2	<0.002	0.25
CD-23–00876	HC150_23594	Human	6	0.5	0.047	1	>32	1.5	0.38	0.5

*CLI, clindamycin; CIP, ciprofloxacin; LIN, linezolid; MET, metronidazole; MOX, moxifloxacin; RIF, rifampicin; TET, tetracycline; VAN, vancomycin.

†Clinical breakpoints are indicated according to CLSI [48].

‡Clinical breakpoints are indicated according to EUCAST (https://www.eucast.org/clinical_breakpoints, https://mic.eucast.org/search/; accessed 22 July 2024).

§Clinical breakpoint is indicated according to Behra-Miellet *et al.* [57].

### Sharing of near-identical genomes across different niches

Hierarchical clustering of cgMLST allelic profiles at level HC2 (i.e. genomes with pairwise distances of ≤2 cgMLST alleles) was previously shown to correlate with core genome SNP analyses and to be consistent with inter-patient transmission [21]. We detected 27 HC2 clusters indicating intra-species, inter-species and inter-farm sharing of closely related genomes (Table S2, sheet 1). Close relatedness was subsequently confirmed by analyses of genome-wide SNPs (Table S2, sheets 2–28).

**Fig. 4.**
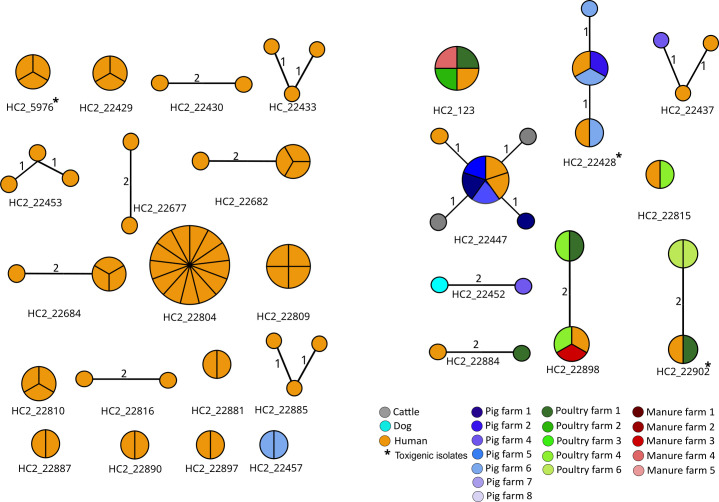
Rapid neighbour-joining phylogenetic trees based on cgMLST allelic distances. Nodes are coloured by isolation source. *C. difficile* isolates from humans had been collected from different patients within the same hospital. The scale bar indicates the branch length corresponding to one cgMLST allelic difference.

*Within hospital, intra- and inter-farm clonal spread (intra-species sharing):* We detected clusters of human–human, pig–pig and poultry–poultry sharing of closely related genomes for both toxigenic (12.5%, *n*=3/24 clusters) and non-toxigenic (87.5%, *n*=21/24 clusters) *C. difficile* strains, including the globally disseminated HC150_1 (RT078), HC150_3 (RT001) and HC150_3622 (RT014). Nineteen different clusters of intra-hospital sharing were detected (Fig. 4). The closely related strains originated from different patients hospitalized in four different wards [medical (i.e. internal medicine), obstetrics/gynaecology, paediatrics and surgical] or visiting the general out-patient clinic of the same hospital, respectively, suggestive of intra-hospital spread. Clusters were spread within the same ward (*n*=4 clusters) and across multiple wards (*n*=4 clusters) (Supplementary Material 1). Similarly, we detected within and between farm sharing of closely related *C. difficile* genomes for both pigs and poultry suggesting clonal dissemination (Fig. 4).

*Inter-species sharing:* We detected clusters of closely related genomes originating from humans, cattle, dogs and pigs (Fig. 4) indicating that *C. difficile* was shared among different host species. Interspecies (inter-source) sharing of near-identical *C. difficile* genomes was also observed between human and poultry, human and pigs, dog and pig, human, poultry and poultry manure for both toxigenic (22%, *n*=2/9 clusters) and non-toxigenic strains (78%, *n*=7/9 clusters) (Fig. 4).

### Few genome sequences have relatives in the public record

Leveraging on the large number of publicly accessible *C. difficile* genomes hosted within EnteroBase, we investigated the genetic relationships of our study genomes within the context of the global *C. difficile* population. For the majority of our genomes (59%, 115/194), there were no related genome sequences at the level HC150 in the public database, indicating that they were genetically distinct from previously published sequences. Accordingly, 79% of the HC150 clusters assigned had no previously published member in the genome database (42/53 for all strains; 54%, 6/11 for toxigenic strains). Actual PCR ribotyping of representative isolates from each of 53 different HC150 clusters confirmed genome-based ribotype predictions (Table 2). For 16 HC150 clusters with no ribotypes assigned, it was revealed that they represented novel PCR ribotypes (Table 2). Since most (83%) of our genome sequences did not carry any *C. difficile* toxin genes, the majority of novel HC150 clusters and novel PCR ribotypes were associated with non-toxigenic isolates. Out of 112 HC2 clusters found in our data from Nigeria, only two (1.8%; HC2_5976 and HC2_123) included relatives in EnteroBase that had been collected from other countries previously.

## Discussion

### Large extant strain diversity of *C. difficile*

Using WGS and phylogenomic analyses of 194 *C*. *difficile* genomes recovered from humans and animals in Makurdi, Nigeria, we show that the circulating strains in our setting comprise both globally disseminated strains and locally distinct HC150 clusters. Consistent with previous studies from Africa, the epidemic RT027 (and related strains, e.g. RT955) were not detected, further corroborating its geographic restriction to other continents [58]. Clinical diagnosis of CDI, although rare in our setting, is often based on the presentation of symptoms including diarrhoea, detection of the toxin or isolation of *C. difficile* from the patient’s faeces [59]. In countries with high-income economies, CDI is a disease primarily of the elderly [60,68], with advanced age (≥65 years) identified as an important risk factor for the onset of CDI [59,64,66, 69, 70]. On the contrary, and consistent with our findings, reports particularly from Africa have diagnosed CDI and recovered *C. difficile* from younger persons [13,14, 16, 50, 51, 71]. These observations have been attributed partially to the population demography in Africa, which is tilted towards a predominance of younger people [12,13, 51, 71]. It could also be a result of the increased healthcare seeking and accessibility of the younger population. Nevertheless, these observations of differing CDI epidemiology in Africa warrant in-depth follow-up studies. Diagnosis or treatment of *C. difficile* is not routinely carried out in the hospitals that we have investigated here, as is the case all over the country. As a consequence, the true burden and incidence of *C*DI in Nigeria are unknown. Even though other pathogens could not be entirely excluded, co-infections with *C. difficile* and *Escherichia coli* or *S. enterica* were observed very rarely in the present study. Recovery from the diarrhoeic patients of toxigenic *C. difficile* strains, particularly HC150_3 (RT001), HC150_3622 (RT014) and HC150_1 (RT078) which have been associated with human disease previously [22,26, 27, 72], strongly suggests that these patients were suffering from CDI. Hence, our results infer that CDI may require increased recognition in Africa, contrary to the assumption that it may not be much of a problem on the continent. HC150_3 (RT001) and HC150_3622 (RT014) have been previously reported from dogs in Nigeria [18], and our detection of these strains in animal and human populations further asserts their broad host range [27].

### The *C. difficile* population in Nigeria includes many unique genotypes

The majority of *C. difficile* strains had no genetic relatives (at the HC150 level) in the public database and therefore appeared to be unique to Nigeria, which was subsequently confirmed by PCR ribotyping. Our results corroborate that PCR ribotypes can be reliably predicted from genome sequencing data [21,22, 73,75], even though for RT084, clustering at level HC200 appeared more appropriate than the previously suggested level HC150 [21].

While novel RTs are reported sporadically [7,8, 14, 18], the large proportion of unique genotypes observed in the present study is likely caused by under-sequencing from the African continent. The detection of unique toxigenic strains highlights local evolution and the need for increased surveillance in regions currently underrepresented in the pathogen genomic databases to understand regional population structures and their impact on global *C. difficile* epidemiology. In addition, a general bias towards sequencing of toxigenic strains [76] may account for the absence of relatives for our non-toxigenic isolates.

Even though the observed clustering between seven genomes from Nigeria with database entries from other countries at the level HC2 may suggest limited long-distance spread, which could be connected to international human traffic and trade [26,27], this conclusion would require additional epidemiological information.

### The animal husbandry system provides a reservoir for *C. difficile* spread

The detection of clonal intra-species, inter-species and inter-farm sharing of near identical genomes suggests that *C. difficile* may be spreading unnoticed within hospitals, between farms and between animals and humans. We hypothesize that the unique livestock production system and poor to nonexistent biosecurity practices in this setting may be partly driving the observed farm-farm and animal-human transmissions since it provides increased opportunities for frequent human-animal contact. Indirect transmission of *C. difficile* could be facilitated by contamination of the environment [22] or food [77,78]. Further, trading and movement of livestock, including poultry and pigs, have been identified as an important pathway for disease spread in Nigeria [79,80]. Similarly, genetically identical *Salmonella* isolates have been recovered from day-old birds and from the source hatcheries, suggesting that the breeding flocks may be the source for the spread of the pathogen to farms in Nigeria [81]. In our study setting, birds are often pre-ordered from multiple hatcheries across the country and then trucked into the state. Three collection points exist for offloading of these birds by the transporters and for easy pickup by the farmers. Similarly, major sources of pigs for farm stocking in our study setting are live pig markets and purchase from fellow farmers. The pig market days in particular often witness aggregation of a large number of pigs (400–6000) at the same time, often with a commingling of the animals [79]. This practice of congregating large numbers of animals from different sources provides an interface and increased opportunity for the spread and exchange of pathogens including *C. difficile*, which are then disseminated to farms, and may also account for the apparent clonal spread observed within and between farms. However, this pathway will require further investigation by the sampling of animals at points of embarkment (hatchery or farm) and dis-embarkment, or upon arrival at the farms, to ascertain their *C. difficile* colonization status. Transportation from one part of the country to another may also result in countrywide spread since these animals are transported via road in open vehicles crisscrossing different states. Long-range disseminations of *C. difficile* spores due to animal and human movement have been previously suspected [27]. The poultry and pig distribution trade network across Nigeria is highly complex, semi-regulated and understudied [79,82]. It would be interesting to investigate how these practices contribute to the spread of zoonotic pathogens including *C. difficile* across the country.

Intriguingly, among the potential transmission clusters is the intra-hospital (human–human) and human–pig sharing of the globally disseminated epidemic *C. difficile* HC150_3622 (RT014) and HC150_1 (RT078) strains. Similar intra-hospital, inter-species and intra-species outbreaks have been reported elsewhere [8,26, 27]. Our findings suggest that there were unnoticed outbreaks and that the region may not be as impervious to *C. difficile* outbreaks as previously thought. They also highlight the need for increased awareness of *C. difficile* among clinicians in Africa.

### Antimicrobial resistance reflects antibiotic consumption

Antimicrobial use and resistance are recognized drivers of *C. difficile* emergence and dissemination [83,84]. Although *C. difficile* antimicrobial susceptibility data from Africa are scarce, reports available from the continent are consistent with our findings of a high prevalence of fluoroquinolone and erythromycin resistance, and a widespread susceptibility to fidaxomicin, metronidazole and vancomycin [8,10, 13, 14, 52]. A contrasting, recent report of the high prevalence of vancomycin and metronidazole resistance in Kenya [85] has been challenged [86,87]. The distributions of resistance determinants in an area are often influenced by its local antimicrobial selection pressures [88,89]. Similarly, the frequency of fluoroquinolone and erythromycin resistance phenotypes and determinants observed for our isolates potentially reflects the antimicrobial consumption pattern and selection pressure in our setting. In Nigeria, fluoroquinolones and macrolides are widely used in human and veterinary medicine, empirically or self-prescribed in cases of enteric infections [90,95]. Similar to our observation on *C. difficile*, resistance to these agents is common in other enteric pathogens across Nigeria, including *Salmonella*, *E. coli* and *Klebsiella* species [81,96,99]. This calls for improved antimicrobial stewardship and public enlightenment on the dangers of empirical and self-prescription [100]. Surprisingly, despite the heavy use of fluoroquinolones, tetracyclines and macrolides in poultry production in our setting, most of the poultry isolates did not express or harbour determinants of resistance. Although the reason for this is not known and may require further investigation, a similar finding has been reported previously for *C. difficile* from poultry in Germany [22].

## Conclusion

We present, to our knowledge, the first study to provide a dataset of *C. difficile* genome sequences (*n*=194) from the African continent, revealing a large number of unique genotypes. The extremely high degree of relatedness between *C. difficile* isolates from different sources suggested intra-species and inter-species clonal spread in the region and highlighted the link between livestock production and human CDI epidemiology. These findings reinforce the need for a One Health approach [101,102] in pathogen surveillance. Further studies are required to understand the reservoirs of *C. difficile* and to reduce its spread.

## Supplementary material

10.1099/mgen.0.001342 Uncited Supplementary Material 1.

10.1099/mgen.0.001342 Uncited Table S1.

10.1099/mgen.0.001342 Uncited Table S2.

## Abbreviations

ANI: average nucleotide identity
CDI: *Clostridioides difficile* infection
cgMLST: core-genome multilocus sequence typing
CLSI: Clinical Laboratory and Standards Institute
EUCAST: European Committee on Antimicrobial Susceptibility Testing
NCBI: National Center for Biotechnology Information
NJ: neighbour-joining
RT: PCR ribotype
