# Supplementary material for: Clostridioides difficile recovered from hospital patients, livestock and dogs in Nigeria share near-identical genome sequences
Source: Microb Genom. 2025 Jan 30;11(1):001342. doi: 10.1099/mgen.0.001342 (PMC12453408; doi:10.1099/mgen.0.001342)
Supplement: Uncited Supplementary Material 1. [file mgen-11-01342-s001.pdf]

Supplementary Materials

Supplementary Figure S1: Rapid-neighbor-joining phylogenetic tree based on cgMLST allelic distances, as in Figure 1. In panel A, color labelling indicates the different HC150 clusters. In panel B, color labelling indicates the sources of toxigenic strains, whereas white nodes indicate non-toxigenic strains.

Suppl. Table S1, sheet 1: Metadata for 194 genome sequences.

Suppl. Table S1, sheet 2: Average nucleotide identities (ANI) among cryptic clade genomes.

Suppl. Table S1, sheet 3: Allelic profiles of the 7-gene MLST for cryptic clade isolates.

Suppl. Table S2, sheet 1: List of genetic clusters of closely related genomes.

Suppl. Table S2, sheets 2-28: Recombination-corrected pairwise SNP distances among genomes from HC2 clusters.

Suppl. Table S3: Distribution of closely related (HC2) strains across the different hospital wards and the general out-patient clinic.

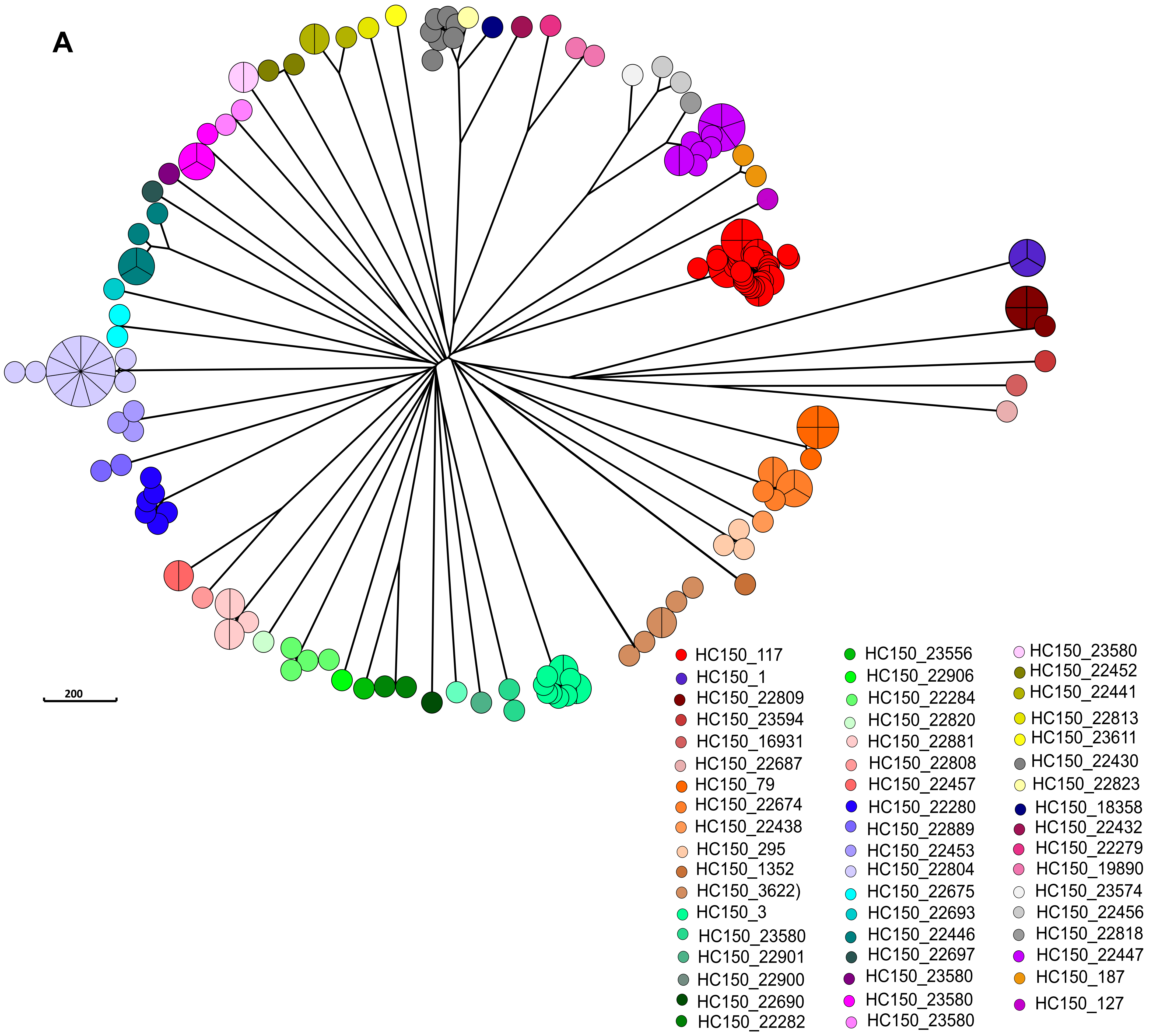
A
200
HC150_117
HC150_1
HC150_22809
HC150_23594
HC150_16931
HC150_22687
HC150_79
HC150_22674
HC150_22438
HC150_295
HC150_1352
HC150_3622)
HC150_3
HC150_23580
HC150_22901
HC150_22900
HC150_22690
HC150_22282
HC150_23556
HC150_22906
HC150_22284
HC150_22820
HC150_22881
HC150_22808
HC150_22457
HC150_22280
HC150_22889
HC150_22453
HC150_22804
HC150_22675
HC150_22693
HC150_22446
HC150_22697
HC150_23580
HC150_23580
HC150_23580
HC150_23580
HC150_22452
HC150_22441
HC150_22813
HC150_23611
HC150_22430
HC150_22823
HC150_18358
HC150_22432
HC150_22279
HC150_19890
HC150_23574
HC150_22456
HC150_22818
HC150_22447
HC150_187
HC150_127

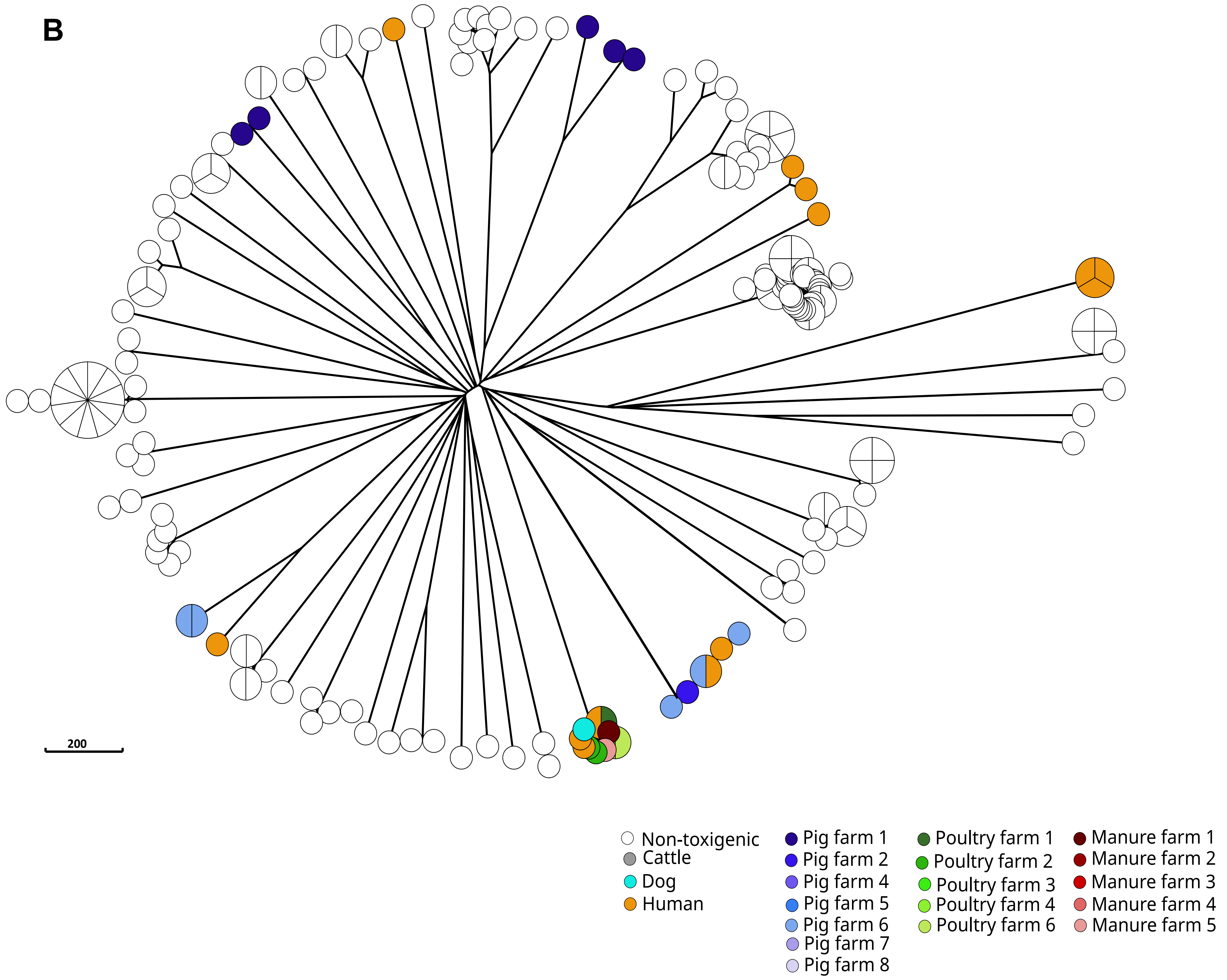
B
200
Non-toxigenic
Cattle
Dog
Human
Pig farm 1
Pig farm 2
Pig farm 4
Pig farm 5
Pig farm 6
Pig farm 7
Pig farm 8
Poultry farm 1
Poultry farm 2
Poultry farm 3
Poultry farm 4
Poultry farm 6
Manure farm 1
Manure farm 2
Manure farm 3
Manure farm 4
Manure farm 5

Table S3: Distribution of closely related (HC2) strains across the different hospital wards and the out-patient clinic.

**HC2 cluster**	**HC150 (ribotype)**	**Number of human isolates in the cluster**	**Number of isolates within the cluster emanating from the respective wards**				
			**Out-patient clinic**	**Medical**	**Obstetrics & gynaecology**	**Paediatrics**	**Surgical**
HC2_5976	HC150_1 (RT078)	3	-	-	-	-	3
HC2_22428	HC150_3622 (RT014)	2	-		-	-	2
HC2_22429	HC150_117 (RT084)	4	4	-	-	-	-
HC2_22430	HC150_22430 (RT713)	2	-	-	-	-	2
HC2_22433	HC150_117 (RT084)	3	2	-	1	-	-
HC2_22437	HC150_22284 (RT681)	2	1	1	-	-	-
HC2_22447	HC150_22447 (novel RT)	3	2	1	-	-	-
HC2_22677	HC150_22430 (RT713)	2	-	2	-	-	-
HC2_22682	HC150_117 (RT084)	4	2	1	-	-	1
HC2_22684	HC150_22684 (RT011)	4	-	4	-	-	-
HC2_22804	HC150_22804 (RT056)	13	2	4	-	1	6
HC2_22809	HC150_22809 (novel RT)	4	-	-	1	3	-
HC2_22810	HC150_22446 (RT535)	3	1	1	1	-	-
HC2_22816	HC150_22675 (RT1059)	2	-	2	-	-	-
HC2_22881	HC150_22881 (RT019)	2	-	2	-	-	-
HC2_22885	HC150_117 (RT084)	3	-	-	-	3	-
HC2_22887	HC150_117 (RT084)	2	2	-	-	-	-
HC2_22890	HC150_22447 (novel RT)	3	-	-	1	1	1
HC2_22897	HC150_22881 (RT019)	2	-	-	-	2	-

RT = ribotype
